# Serum homocysteine concentration as a marker for advanced diabetic nephropathy in a cohort of elderly patients

**DOI:** 10.1186/s12902-023-01342-1

**Published:** 2023-05-22

**Authors:** Xulei Zheng, Qiaorui Liu, Zhiwen Liu

**Affiliations:** 1 Department of Endocrinology, Shanghai Xuhui Central Hospital/Xuhui Hospital, Fudan University, 966 Huaihai Middle Road, Xuhui District, Shanghai, 200031 China; 2 Inpatient department, Jiahui International Hospital (Shanghai), 689 Guiping Road, Xuhui District, Shanghai, 200233 China

**Keywords:** Diabetic nephropathy, Glomerular filtration rate, Homocysteine, Urinary protein/creatinine ratio, Vitamin D

## Abstract

**Background:**

Hyperhomocysteinemia has been linked with chronic kidney disease (CKD). The present study investigated whether homocysteine (Hcy) serum levels might serve as a marker for the advancement of diabetic nephropathy (DN).

**Methods:**

Clinical and laboratory indicators including Hcy, vitamin D (VD), urine protein, estimated glomerular filtration rate (eGFR) and the urinary protein/creatinine ratio in subjects > 65 years with DN (n = 1,845), prediabetes (n = 1,180) and in a non-diabetes (control) group (n = 28,720) were analyzed.

**Results:**

DN patients had elevated Hcy concentrations, decreased VD and higher urinary protein levels, a reduced eGFR and a higher urinary protein/creatinine ratio compared with prediabetic and control subjects. After correcting for urinary protein quantitation, multivariate analysis revealed that both the Hcy concentration (*P* < 0.010) and urinary protein/creatinine ratio (*P* < 0.001) were risk factors, while the VD2 + VD3 serum concentration (*P* < 0.001) was a protective factor for DN. Moreover, Hcy > 12 µmol/L was a cut-off value for predicting advanced DN.

**Conclusion:**

Hcy serum concentration is a potential marker for the advancement of CKD in DN but not prediabetes patients.

## Introduction

The incidence of diabetes is about 8.8% for individuals aged between 20 and 79 years and affected circa 440 million people globally in 2015, with the numbers projected to increase to > 550 million by 2035 [1]. Risk factors associated with diabetic nephropathy (DN) include dyslipidemia, hypertension, smoking, as well as the degree of glycemic control. Unmodifiable risk factors include age, ethnicity, genetic profiles [2], with hypertension and hyperglycemia being particularly important [3]. DN is characterized by a marked increase in the excretion of albumin in the urine, diabetic glomerular lesions and a reduced estimated glomerular filtration rate (eGFR) in patients with diabetes [2]. However, in the elderly population, a decline of eGFR is a normal physiologic process due to increased glomerulosclerosis and decreased functioning nephron numbers [4]. In addition, another study noted that 20% of type 2 diabetes mellitus (DM) patients exhibit early progressive eGFR decline with normoalbuminuric and the need of new markers apart from albuminuria has been underlined for these kinds of progressive CN patients [5].

Homocysteine (Hcy) is an amino acid that is produced by the metabolism of the amino acid methionine [6] and elevations of serum Hcy concentrations, a condition known as hyperhomocysteinemia [6], has been linked with chronic kidney disease (CKD), particularly in end stage renal disease where Hcy has been associated with a decrease in eGFR [6]. Other studies reported controversial results and the role of Hcy in the development of DN has not yet been unequivocally determined [7].

Vitamin D (VD) may have actions in the development of DN [8]. The pathogenesis of DN is known to be associated with inflammation and oxidative stress, processes modulated by nuclear VD receptors (VDRs) among others. Moreover, VD and metabolic derivatives have been used to treat patients with CKD, with a fair degree of success [9]. The effects of VD therapy on DN patients, however, have not yet been systematically investigated in the clinic.

Therefore, the aims of the present retrospective cross-sectional study were to evaluate potential relationships between the Hcy serum concentration and VD in addition to other factors in patients with DN, as well as for the first time in China patients with prediabetes We hypothesized that Hcy serum concentrations might be correlated with the advancement of CKD in DN patients and VD might be a potential supplemental therapy for DN treatment.

## Methods

### Ethics approval and informed consent

The study was conducted in strict accordance with the Declaration of Helsinki and was approved by the Ethics Committee of Shanghai Xuhui Central Hospital (No. 2019-092). Informed consent was obtained from all individual participants in the study.

### Criteria for inclusion/exclusion in the study

#### Inclusion criteria

Patients in our hospital with type 2 diabetes and non-diabetes subjects > 65 years old were enrolled from May 2012 to August 2017, since DN incidence and related symptoms are correlating with age [10]. The diagnosis of type 2 diabetes was confirmed using World Health Organization and American Diabetes Association guidelines including a fasting plasma glucose concentration (PGC) > 7.0 mmol/L, a non-fasting PGC > 11.1 mmol/L, a 2-hour PGC > 11.1 mmol/L in an oral glucose tolerance test or the use of blood glucose lowering drugs. The glycosylated hemoglobin (HbA1c) concentration was not considered as a diagnosis criteria in this study because the test used in our hospital’s laboratory were not National Glycohemoglobin Standardization Program certified. However, HbA1c concentrations were taken into account in our analysis of the variables measured.

Non-diabetes patients consisted of health checked subjects who did not have type 2 diabetes.

Diabetic kidney disease was diagnosed in patients with an obvious clinical history of type 2 diabetes, with a number of laboratory measurements made < 6 months apart showing a urinary albumin-to-creatinine ratio ≥ 30 mg/g, a urinary albumin excretion rate ≥ 30 mg/24 hours and/or eGFR < 60 mL/min for > 3 months confirming the diagnosis. Impaired fasting glucose and abnormal glucose tolerance were considered to reflect prediabetes (see World Health Organization criteria for details).

#### Exclusion criteria

Patients treated with hormones; liver failure; a serum creatinine > 120 mmol/L; hypo- or hyperparathyroidism; immune system deficiency; a body mass index ≥ 30 kg/m^2^; infectious disease; or congenital disorders of metabolism were excluded from the study.

Study design.

In this retrospective cross-sectional study, we first accessed our hospital’s Health Information System (HIS) database and screened all patients who came to our hospital between 2012 and 2017. The enrolled patients were over 65 years of age according to the inclusion criteria, and we focused on the analysis of Hcy, VD, urine protein, estimated glomerular filtration rate (eGFR) and the urinary protein/creatinine ratio, and DN was diagnosed. Other diagnoses included prediabetes without nephropathy, diabetes without nephropathy and non-diabetics (controls).

### Analysis of serum samples

Total VD was measured as the sum of the serum concentrations of VD and its main metabolites with an API LC-MS/MS system (AB Sciex Pte Ltd, US). The internal standards were deuterated 6, 19, 19-d_3_ and 26, 26, 26, 27, 27 and 27-d_6_ (MilliporeSigma, US) and metabolite concentrations were measured in the range 2.5–200 ng/mL. Hcy was measured using LC-MS/MS as previously described [11]. The resulting data were evaluated using Analyst 1.5 software (Applied Biosystems, US). Renal and serum lipid concentrations were measured by employing an Advia Clinical Chemistry System (Siemens Healthcare, Germany).

### Statistical analysis

IBM SPSS Statistics for Windows (ver. 20.0. Armonk, NY) was employed for all data analyses. As a retrospective study, the results were based on analysis of previous data (that met the present study’s exclusion/inclusion criteria) on the effects of VD on lymphocyte counts. The cohort size was therefore not specifically estimated. Continuous data with a normal distribution are reported as the mean ± SD, while discrete data are given as a number or percentage and a χ^2^ test was employed to investigate potential intergroup differences. Any intergroup differences in continuous data were analyzed using a *t*-test. Non-normally distributed continuous variables were first logarithmically transformed before evaluation. The lymphocyte population was distributed normally as revealed by the Kolmogorov-Smirnov test. ANOVA was used to compare 3 groups. If the overall differences were significant, the Student-Newman-Keuls method was employed for pairwise comparisons. For subgroup analysis of Hcy concentrations, based on the immune cell marker results with different serum total VD concentrations, ANOVA was used after adjustments were made for age and sex, and HbA1, triglyceride and cholesterol concentrations. A *P*-value < 0.05 was deemed to be a significant finding.

## Results

### Demographic characteristics of enrolled subjects

All the subjects were selected from a large database of real-world studies. A total of 31,745 people were enrolled, with an average age of 75.9 years. They were divided into a non-diabetes group of 28,720 subjects (90.5%), a prediabetes group of 1,180 subjects (4.1%) and a DM group comprised of 3,025 subjects (9.5%), of whom 476 (15.7%) had diabetic retinopathy and 1,845 (61%) DN, with various comorbidities such as hypertension (55.2%), coronary heart disease (23.4%) and peripheral vascular disease (41.5%). It is worth noting that almost 50% of the DN patients had three types of complications, implying that DN can trigger many other pathological conditions. In addition, 704 (23.3% diabetics without nephropathy were included in the analysis (Table 1)).

**Table 1:** Patients’ characteristics at baseline

	N	%
**Total subjects**	31,745	
**Age (year)**	75.9 ± 14.5	
**Non-diabetes**	28,720	90.5
Prediabetes	1,180	4.1
**DM**	3,025	9.5
Diabetic retinopathy	476	15.7
DN	1,845	61.0%
DN + hypertension	1,018	55.2
DN + coronary heart disease	432	23.4
DN + peripheral vascular disease	766	41.5
DN with complications		
DN + ≤ 2 types of complications	90	4.9
DN + 3 types of complications	875	47.4
DN + 4 types of complications	732	39.7
DN + 5 types of complications	148	8.0
Diabetics without nephropathy	704	23.3
**Medication for DN**	1845	100
Insulin	915	49.6
Glinides	373	20.2
α-glucosidase inhibitor	356	19.3
Others	201	10.9
**Medication for prediabetes**	1180	100
Metformin	635	53.8
α-glucosidase inhibitor	277	23.5
Thiazolidinedione	140	11.9
Others	128	10.8

Abbreviations: DM, diabetes mellitus; DN, diabetic nephropathy; N, number

The subjects clinical and laboratory indicators were also compared between the three groups (all *P*-value < 0.05). Compared with prediabetic patients, DN patients had higher serum Hcy concentrations, lower VD concentrations, higher urinary protein levels, lower eGFRs, and higher urinary protein/creatinine ratios (all *P* < 0.001). For prediabetes subjects, urinary protein/creatinine values and fasting blood glucose values were above the normal range, while in the non-diabetic control group all data were in the normal health range (Table 2).

**Table 2:** Patient’s clinical and laboratory indicators at baseline

Variable	DN (N = 1,845)	Prediabetes (N = 1,180)	Non-diabetes (N = 28,720)	*P*-value (DN vs. Prediabetes)	*P*-value (DN vs. non-diabetes)	*P*-value (Prediabetes vs. non-diabetes)
Age (years)	73.0 (64.0–84.0)	84.0 (67.0–91.0)	80.0 (66.0–87.0)	< 0.001	< 0.001	< 0.001
BMI (kg/m^2^)	27.5 ± 4.1	26.8 ± 3.8	26.3 ± 3.5	< 0.001	< 0.001	< 0.001
SBP (mmHg)	140.2 ± 21.5	134.1 ± 20.3	132.3 ± 19.2	< 0.001	< 0.001	0.002
DBP (mmHg)	83.6 ± 14.5	80.4 ± 13.5	79.9 ± 12.3	< 0.001	< 0.001	0.173
BUN (mmol/L)	5.2 ± 2.1	5.0 ± 1.5	5.0 ± 1.1	< 0.001	< 0.001	1.000
Cr (µmol/L)	75.3 ± 17.5	72.3 ± 18.2	71.8 ± 17.2	< 0.001	< 0.001	0.329
HbA1c (%)	8.9 (2.3–15.2)	6.1 (1.8–11.2)	5.1 (1.2–9.5)	< 0.001	< 0.001	< 0.001
1,5-AG (mg/L)	2.8 (1.5–3.5)	8.9 (5.7–10.2)	9.2 (6.3–18.6)	< 0.001	< 0.001	< 0.001
Blood glucose index (mmol/L)	12.8 (7.8–19.0)	7.8 (5.8–10.8)	5.9 (4.2–6.5)	< 0.001	< 0.001	< 0.001
Hcy (µmol/L)	16.5 (12.3–23.0)	12.0 (9.9–15.3)	14.1 (11.0–19.2)	< 0.001	< 0.001	< 0.001
VD2 (ng/mL)	0.5 (0.0–1.4)	0.9 (0.2–1.8)	0.7 (0.0–1.7)	< 0.001	< 0.001	0.034
VD3 (ng/mL)	11.2 (7.3–16.2)	14.1 (8.9–21.4)	12.5 (8.4–18.1)	< 0.001	< 0.001	< 0.001
VD2 + VD3 (ng/mL)	12.3 (8.3–18.0)	16.6 (11.4–22.8)	14.2 (9.7–20.4)	< 0.001	< 0.001	< 0.001
eGFR (mL/min)	38.0 (18.0–67.9)	77.8 (59.4–98.3)	74.6 (55.4–94.0)	< 0.001	< 0.001	<0.001
Urine protein quantification (g/24 h)	0.7 (0.2–2.5)	0.1 (0.0–0.1)	0.1 (0.1–0.6)	< 0.001	< 0.001	< 0.001
Urinary protein/creatinine (mg/mmol)	386.4 (90.5–1,884.0)	27.8 (9.4–146.4)	20.8 (8.8–82.0)	< 0.001	< 0.001	0.004

Notes: Data are given as the median (IQR)

Abbreviations: 1,5-AG, 1,5-anhydro-d-glucitol; DN, diabetic nephropathy; eGFR, estimated glomerular filtration rate; HbA1c, glycosylated hemoglobin; Hcy, homocysteine; N, number; VD, vitamin D

### Univariate and multivariate analysis of DN risk factors

Univariate regression analysis revealed that age, Hcy and VD concentrations, urinary protein levels, eGFR, and the ratio of urinary protein/creatinine were all significant risk factors for DN patients. Multivariate regression analysis revealed that only age and urine protein quantification were risk factors and that VD2 + VD3 was a protective factor for DN (Table 3). As urinary protein quantification itself is a clinical manifestation of nephropathy, it will lead to no statistical significance of other variables as a risk factor. Therefore, we made an adjustment to correct the urinary protein quantitation in multivariate analysis. The analysis revealed that both Hcy and urinary protein/creatinine ratio were, in addition to eGFR, risk factors, while VD2 + VD3 was still a protective factor for DN. In addition, a concentration of Hcy > 12 µmol/L was a high risk factor for DN, suggesting the importance of serum Hcy concentrations in DN patients as a cut-off value (Table 3).

**Table 3:** Univariate and multivariate analyses of risk factors for DN

	Univariate analysis		Multivariate analysis			
Indicators	OR (95% CI)	*P*-value	Before adjusting		After adjusting*	
			OR (95% CI)	*P*-value	OR (95% CI)	*P*-value
Age (years)	0.97 (0.97–0.98)	< 0.001	0.96 (0.94–0.97)	< 0.001	1.07 (1.04–1.10)	< 0.001
Hcy (µmol/L)	1.10 (1.08–1.12)	< 0.001				
Hcy < 12 (µmol/L)	1.00					
Hcy 12–19 (µmol/L)	2.35 (1.86–2.98)	< 0.001			1.92 (1.27–2.92)	0.002
Hcy > 19 (µmol/L)	5.76 (4.31–7.71)	< 0.001			3.28 (1.81–5.96)	< 0.001
VD2 (ng/mL)	0.94 (0.91–0.97)	<0.001				
VD3 (ng/mL)	0.96 (0.95–0.97)	< 0.001				
VD2 + VD3 (ng/mL)	0.95 (0.94–0.97)	< 0.001	0.95 (0.94–0.97)	< 0.001	0.97 (0.94–0.99)	0.048
Urine protein quantification (mg/24 h)	108.93 (21.36–555.41)	< 0.001			19.74 (4.38–88.95)	< 0.001
eGFR (mL/min)	0.99 (0.99–0.99)	< 0.001	0.99 (0.98–1.00)	0.002		
Urinary protein/creatinine (mg/mmol)	1.00 (1.00–1.00)	< 0.001	1.00 (1.00–1.00)	< 0.001		

Note: * adjusted for urine protein quantitation

Abbreviations: CI, confidence interval; eGFR, estimated glomerular filtration rate; Hcy, homocysteine; OR, odds ratio; VD, vitamin D

### Hcy concentration stratification analysis

To evaluate further the effect of serum Hcy concentrations in DN patients, we stratified all subjects into 3 groups (Hcy concentrations < 12 µmol/L, 12–19 µmol/L and > 19 µmol/L groups, respectively) and compared the clinical indicators among them. It was found with an increase in serum Hcy concentrations the concentration of VD2 and VD3 decreased gradually. Similarly, an increase of serum Hcy, urinary protein quantification and the urinary protein/creatinine ratio, which are related to nephropathy, increased rapidly and eGFR was significantly decreased (Table 4). Therefore, an increased Hcy concentration appears to be a good indicator to predict kidney disease, and it may be desirable to control the concentration of Hcy in prediabetes subjects or DN patients.

**Table 4:** Analysis of clinical indicators stratified according to the serum Hcy concentration

	Hcy (µmol/L)			*P*-value
	< 12	12–19	> 19	
Number of subjects	N = 9,988	N = 12,565	N = 7,815	
VD2 (ng/mL)	1.90 ± 4.01	1.74 ± 3.74	1.43 ± 2.73	< 0.001
VD3 (ng/mL)	14.54 ± 8.08	13.91 ± 7.92	13.07 ± 7.70	< 0.001
VD2 + VD3 (ng/mL)	16.46 ± 8.46	15.63 ± 8.43	14.47 ± 8.15	< 0.001
Urine protein quantitation (g/24 h)	0.66 ± 1.58	1.01 ± 1.90	1.28 ± 1.86	< 0.001
eGFR (mL/min)	88.84 ± 28.07	72.97 ± 26.00	59.01 ± 28.64	< 0.001
Urinary protein/creatinine (mg/mmol)	167.45 ± 678.65	288.48 ± 1,585.37	399.09 ± 1,066.41	< 0.001

Abbreviations: eGFR, estimated glomerular filtration rate; Hcy, homocysteine; N, number; VD, vitamin D

### Correlation analysis

A Spearman correlation analysis was performed to evaluate the relationship between the serum Hcy concentration and urine protein quantitation, or between Hcy and eGFR in DN patients, in prediabetes subjects or in the DN and prediabetes population. The serum concentration of Hcy in DN was directly proportional to the urinary protein quantification (r = 0.112, *P* = 0.004) and inversely proportional to eGFR (r = -0.425, *P* < 0.001) (Table 5). However, there was no correlation between the serum Hcy concentration and urinary protein quantification in prediabetes subjects (r = 0.124, *P* = 0.355), while the serum Hcy concentration was negatively correlated with the eGFR values (r = -0.135, *P* = 0.002). These results suggested that the elevation of the serum Hcy concentration is a potential biomarker for nephropathy of diabetic patients, which would be a convenient easily measured predictor in serum to use for advance DN diagnosis.

**Table 5:** Bivariate correlations between serum Hcy concentrations and urine protein quantitation as well as EGFR in different study groups after Spearman correlation analysis

	Urine protein quantification (g/L)			eGFR (mL/min)		
	N	Correlation coefficient	*P*-value	N	Correlation coefficient	*P*-value
Hcy (µmol/L) in DN and prediabetic patients	711	0.106	0.004	1,525	-0.419	< 0.001
Hcy (µmol/L) in DN patients	653	0.112	0.004	983	-0.426	< 0.001
Hcy (µmol/L) in prediabetic patients	58	0.124	0.355	542	-0.135	0.002

Abbreviations: eGFR, estimated glomerular filtration rate; DN, diabetic nephropathy; Hcy, homocysteine; N, number

## Discussion

In the literature, changes in Hcy are mainly caused by mutations of cystathionine-β-synthase (CBS) and methylenetetrahydrofolatereductase (MTHFR), but renal failure also leads to elevated Hcy serum concentrations since the major clearance route from plasma is the kidney. Hcy concentrations were found to be higher in chronic renal disease patients and since Hcy is also a risk factor for atherothrombotic vascular diseases, the high incidence of vascular complications in chronic renal failure patients might be caused by serum Hcy elevations [12]. However, the role of Hcy in the development of DN is considered to date not to be unequivocally established [7]. On the other hand, various studies have found that C677T polymorphism of the MTHFR gene was associated with an increased DN susceptibility, which was most obvious in Asians [13], where the incidence of this mutation has been estimated to be 20.8% [14]. However, since a clear correlation between serum Hcy elevations as a cause for DN has not yet been established, the predisposition to increased plasma Hcy concentrations might only become obvious in individuals with impaired kidney functions and a decline in eGFR [15]. In the present study, a large number of patients with DN were enrolled to evaluate the relationship between the serum Hcy concentration and DN. Our results showed that DN patients had elevated Hcy serum concentrations, decreased VD concentrations, higher urinary protein content, reduced eGFR and higher urinary protein/creatinine ratios compared to prediabetic patients. In addition, after correcting for urinary protein, multivariate analysis revealed that both Hcy and the urinary protein/creatinine ratio were risk factors, while VD2 + VD3 was a protective factor for DN. Moreover, we found for the first time that a concentration of Hcy > 12 µmol/L was a good indicator to predict impaired kidney function in DN patients. We also found that the serum concentration of Hcy in DN was directly proportional to urinary protein quantification and inversely proportional to the eGFR; in prediabetes patients, Hcy did not play a role.

Various mechanisms are believed to mediate the pathogenic actions of Hcy on the kidney including local oxidative and endoplasmic reticulum stress, hypomethylation and inflammation [16]. Despite the huge amount of evidence that Hcy is toxic, the negative interventional trials led the scientific community to consider Hcy may be a good treatment target [17]. For example, a clinical trial that studied 2,056 patients with advanced CKD showed that therapy with high doses of folic acid and/or various VB analogues had no effects on survival times or in reducing the incidence of cardiovascular complications [18]. However, new evidence has shown that low-dose folic acid is effective in slowing down the progression of CKD [17].

The present study reflects the real situation of diabetes and other comorbidities among elderly patients in Shanghai. Multivariate analysis revealed that a Hcy concentration > 12 µmol/L is a significant independent factor for DN progression in elderly Chinese patients, a finding consistent with those reported in previous studies. For example, a cross-sectional hospital-based Chinese study that enrolled 183 patients with type 2 diabetes showed that the total serum concentration of Hcy was independently associated with the occurrence of early DN [19]. A prospective observational study of 208 patients and 49 non-diabetes controls supported the assertion that the serum Hcy concentration was an early predictor for the progression of DN in patients with type 2 diabetes [20]. Another study compared the effects of Hcy in 55 type 2 diabetes patients with DN and 51 type 2 diabetes patients without DN. The results demonstrated an obviously elevated serum concentration of Hcy in patients with DN compared to the non-diabetic control group. The concentration was also significantly correlated with the severity of renal damage, and was proposed to be a serological marker for early DN diagnosis [21]. A study involving elderly Chinese type 2 diabetes patients found that serum Hcy concentrations were elevated and could potentially serve as a risk factor for diabetic kidney disease, although the serum concentration of Hcy was not considered to be a good biomarker [22].

All these findings raise another question, namely whether the occurrence of DN in the elderly can be effectively reduced by appropriately reducing the serum Hcy concentration. This question should be rigorously investigated in a subsequent study. Our results also indicated that VD2 + VD3 is a protective factor for DN. Renoprotective effects elicited by VD through VDRs are well known and an independent link has been reported between VD deficiency or insufficiency in patients with nephropathy, even after adjusting for ethnicity and various other factors [8]. VD is known to play important roles in the regulation of serum phosphorus and calcium concentrations and these ions are involved in the interaction of VD with its VDRs, which acts on the transcriptional regulation of various genes. Much evidence has accumulated that the VD/VDRs signaling pathway confers kidney-protective actions in DN patients, such as antifibrosis effects, the prevention of injury to podocytes, proteinuria and albuminuria reduction and minimizing inflammation in DN and CKD patients [23, 24]. Another systematic meta-analysis reported that for DN patients, VD supplementation provided beneficial effects on 24-hour protein and inflammation urine indices, but had no effects on serum creatinine, eGFR or glycemic control indices [25]. Therefore, further studies are necessary to provide conclusive evidence about the effects of VD in improving kidney functions, especially in DN patients.

The limitations of the present study were, besides its retrospective design, that the fasting or non-fasting Hcy state, dietary patterns, medication and vitamin supplementation as well as smoking and exercises were not included in the analyses. In addition, the stages of CKD (G1-G5) were not evaluated and should be considered in future studies.

## Conclusion

The serum concentration of Hcy was directly proportional to the urinary protein quantification and inversely proportional to eGFR only in DN cases, but not in prediabetic subjects. These findings suggest that the elevation of the serum Hcy concentration with a threshold of > 12 (µmol/L) might be a potential biomarker for the diagnosis of DN in advance of diabetics. VD supplementation might provide a beneficial clinical effect for the treatment of DN.

## Data Availability

The datasets used or analysed during the current study are available from the corresponding author on reasonable request.
